# Improving GPU-accelerated adaptive IDW interpolation algorithm using fast kNN search

**DOI:** 10.1186/s40064-016-3035-2

**Published:** 2016-08-22

**Authors:** Gang Mei, Nengxiong Xu, Liangliang Xu

**Affiliations:** 1 Department of Geological Engineering, Qinghai University, No.251 Ningda Road, Xining, 810016 China; 2 School of Engineering and Technolgy, China University of Geosciences, No.29 Xueyuan Road, Beijing, 100083 China; 3 Institute of Earth and Environmental Science, University of Freiburg, Albertstr.23B, 79104 Freiburg im Breisgau, Germany

**Keywords:** Spatial interpolation, Inverse Distance Weighting (IDW), *k*-nearest neighbors (*k*NN), Graphics Processing Unit (GPU)

## Abstract

This paper presents an efficient parallel Adaptive Inverse Distance Weighting (AIDW) interpolation algorithm on modern Graphics Processing Unit (GPU). The presented algorithm is an improvement of our previous GPU-accelerated AIDW algorithm by adopting fast *k*-nearest neighbors (*k*NN) search. In AIDW, it needs to find several nearest neighboring data points for each interpolated point to adaptively determine the power parameter; and then the desired prediction value of the interpolated point is obtained by weighted interpolating using the power parameter. In this work, we develop a fast *k*NN search approach based on the space-partitioning data structure, even grid, to improve the previous GPU-accelerated AIDW algorithm. The improved algorithm is composed of the stages of *k*NN search and weighted interpolating. To evaluate the performance of the improved algorithm, we perform five groups of experimental tests. The experimental results indicate: (1) the improved algorithm can achieve a speedup of up to 1017 over the corresponding serial algorithm; (2) the improved algorithm is at least two times faster than our previous GPU-accelerated AIDW algorithm; and (3) the utilization of fast *k*NN search can significantly improve the computational efficiency of the entire GPU-accelerated AIDW algorithm.

**Electronic supplementary material:**

The online version of this article (doi:10.1186/s40064-016-3035-2) contains supplementary material, which is available to authorized users.

## Introduction

Spatial interpolation is a fundamental tool in Geographic Information System (GIS). The most frequently used spatial interpolation algorithms include the Inverse Distance Weighting (IDW) (Shepard 1968), Kriging (Krige 1951), Discrete Smoothing Interpolation (DSI) (Mallet 1989, 1992), nearest neighbors, etc; see a comparative survey investigated by Falivene et al. (2010). When applying those interpolation algorithms for large-scale datasets, the computational cost is in general too high (Huang and Yang 2011). A common and effective solution to the above problem is to perform the interpolating in parallel. Currently, a number of research efforts have been conducted to parallelize the spatial interpolation algorithms on various parallel computing platforms (Shi and Ye 2013).

For example, in order to speed up the Kriging interpolation method, Pesquer et al. (2011) designed an effective solution to parallelizing the ordinary Kriging by exploiting the MPI (Message Passing Interface) libraries in a High Performance Computing environment, and significantly improved the computational efficiency of the entire process. Similarly, Strzelczyk and Porzycka (2012) presented a new parallel Kriging algorithm to deal with unevenly spaced data. Cheng (2013) proposed an efficient parallel scheme to accelerate the universal Kriging algorithm on the NVIDIA CUDA platform by optimizing the compute-intensive steps in the Kriging algorithm, such as matrix–vector multiplication and matrix–matrix multiplication and achieved a nearly 18 speedup over the serial program.

Allombert et al. (2014) introduced an efficient out-of-core algorithm that fully benefited from graphics cards acceleration on a desktop computer, and found that it was able to speed up Kriging on the GPU with data four times larger than a classical in-core GPU algorithm, with a limited loss of performances.

To improve the computational efficiency of the most time-consuming steps in ordinary Kriging, i.e., the calculating of weights and then the prediction of each unknown point, Ravé et al. (2014) investigated the potential strategy for reducing the computational cost by by employing suitable operations involved in those steps to be parallelized by using general-purpose computing on GPUs and CUDA.

Hu and Shu (2015) proposed an improved coarse-grained parallel algorithm to accelerate ordinary Kriging interpolation in a homogeneously distributed memory system using the MPI (Message Passing Interface) model and achieved the speedups of up to 20.8. Wei et al. (2015) proposed an algorithm based on the *k*-d tree method to partition a big dataset into workload-balanced child data groups, and achieved high efficiency when the datasets were divided into an optimal number of child data groups.

The IDW interpolation algorithm has been also parallelized on various platforms. For example, exemplified by a hybrid IDW algorithm to generate DEM from LiDAR point clouds, Guan and Wu (2010) designed and implemented a parallel algorithm on multi-core platforms to handle about one billion LiDAR points in approximately 12 min. Huraj et al. (2010a, b) accelerated the IDW method on the GPU for predicting the snow cover depth at the desired point.

Xia et al. (2010, 2011) attempted to map the IDW interpolation to the GPU for parallelization and proposed a GPU-based framework for geospatial analysis, which gave rise to a high computational throughput. Huang et al. (2011) explored of the implementation of a parallel IDW interpolation algorithm in a Linux cluster-based parallel GIS. Li et al. (2014) developed their IDW interpolation application uses the Java Virtual Machine (JVM) for the multi-threading functionality.

Mei (2014) developed two GPU implementations (i.e., the tiled version and the CDP version) of the standard IDW interpolation algorithm by utilizing the shared memory and the feature of CUDA Dynamic Parallelism, and found that the tiled version is about 120 and 670 times faster than the CPU version when the power parameter was specified to 2 and 3.0, respectively. Mei and Tian (2016) also evaluated the impact of several data layouts on the efficiency of GPU-accelerated IDW interpolation.

Some of the other efforts have been also carried out to parallelize other interpolation algorithms. For example, Wang et al. (2010) presented a computing scheme to speed up the Projection-Onto-Convex-Sets (POCS) interpolation for 3D irregular seismic data with GPUs. Guan et al. (2011) developed a parallel the fast Fourier transform (FFT) based geostatistical areal interpolation algorithm in a homogeneously distributed memory system using the MPI programming model. Huang et al. (2012) employed the *k*-d tree in nearest neighbors search to accelerate the grid interpolation on the GPU. Cuomo et al. (2013) proposed a parallel method based on radial basis functions for surface reconstruction on GPU.

The Adaptive IDW (AIDW) is an improved version of the standard IDW Shepard (1968), which was originally proposed by Lu and Wong (2008). In the AIDW it attempts to calculate the power parameter adaptively according to the spatial distribution pattern of the data points, while in the standard IDW the power parameter is a user-specified constant value. Due to the adaptive determination of the power parameter, the AIDW method can achieve much more accurate prediction results than those by the standard IDW.

In our previous work (Mei et al. 2015), we have designed and implemented a parallel AIDW algorithm on a GPU. And we have also evaluated the performance of the parallel AIDW method by comparing its efficiency with that of the corresponding serial one. We have observed that our GPU-accelerated AIDW algorithm can achieve the speedups of up to 400 for one million data points and interpolated points on single precision.

In our previous GPU implementations of the parallel AIDW method, we have found that the most computationally intensive step is the *k* nearest neighbors (*k*NN) search for each interpolated points. We have designed a straightforward method to find the *k* nearest neighboring data points for each interpolated point within a single thread. Although the GPU implementing using our straightforward *k*NN search approach can achieve satisfied computational efficiency, for example, the obtained speedups are about 100–400 on single precision, further performance improvement probably can be achieved by optimizing the *k*NN search.

The task of the *k*NN search is to find the nearest neighbors to an input query. Previous research efforts conducted on the *k*NN search are mainly implemented and optimized on the CPU (Sankaranarayanan et al. 2007). Recently, GPU-accelerated implementations have improved performance by utilizing the massively parallel architecture of a single GPU (Garcia et al. 2008; Leite et al. 2012; Pan and Manocha 2012; Liang et al. 2009; Huang and Yang 2011; Beliakov and Li 2012; Komarov et al. 2014; Liu and Wei 2015), multi-GPUs (Kato and Hosino 2012; Arefin et al. 2012), and GPU clusters (Dashti et al. 2013). Among those GPU-accelerated *k*NN search algorithms, most of them attempt to speed up the brute-force *k*NN search algorithm; and several of them are designed and optimized using space partitioning data structures such as grid (Leite et al. 2012), RP-tree (Pan and Manocha 2012), VP-tree (Liu and Wei 2015), and *k*-d tree (Beliakov and Li 2012).

In this paper, we attempt to improve the efficiency of our previous GPU-accelerated AIDW algorithm by adopting a more efficient *k*NN search approach. The efficient *k*NN search is expected to be performed in a separate stage with the use of the data structure, grid. The resulting values of the *k*NN search are the distances between the *k* nearest neighboring data points to each interpolated point. Those distances are then transferred into another stage of the AIDW to adaptively calculate the power parameter and the expected prediction value (i.e., the weighted average). To evaluate the improved parallel AIDW algorithm, we also compare its efficiency with that of our previous one introduced in Mei et al. (2015).

The rest of this paper is organized as follows. “The AIDW interpolation algorithm” section introduces the background principles of the IDW algorithm, the AIDW algorithm, and the *k*NN search. “The improved GPU-accelerated AIDW method” section describes the strategies and considerations for improving our previous GPU-accelerated AIDW algorithm. “Implementation details” section presents some implementation details of the improved algorithm. Some comparative experimental tests and analysis are provided in “Results and discussion” section. Finally, “Conclusion” section draws several conclusions.

## The AIDW interpolation algorithm

The AIDW is an improved version of the standard IDW (Shepard 1968), which is originated by Lu and Wong (2008). The basic and most interesting idea behind the AIDW is as follows. It adaptively determines the distance-decay parameter $\alpha$ according to the spatial pattern of data points in the neighborhood of the interpolated points. In other words, the distance-decay parameter $\alpha$ is no longer a pre-specified constant value but adaptively adjusted for a specific unknown interpolated point according to the distribution of the nearest neighboring data points.

When predicting the desired values for the interpolated points using AIDW, there are typically two phases: the first one is to determine adaptively the power parameter $\alpha$ according to the spatial pattern of data points; and the second is to perform the weighting average of the values of data points. The second phase is the same as that in the standard IDW.

In AIDW, for each interpolated point, the parameter $\alpha$ can be adaptively determined according to the following steps.

### *Step 1*

 Determine the spatial pattern by comparing the observed average nearest neighbor distance with the expected nearest neighbor distance.

1. Calculate the expected nearest neighbor distance $r_{\exp }$ for a random pattern using:
  1 $$\begin{aligned} r_{\exp } =\frac{1}{2\sqrt{n / A} }, \end{aligned}$$
  where *n* is the number of points in the study area, and *A* is the area of the study region.
2. Calculate the observed average nearest neighbor distance $r_{obs}$ by taking the average of the nearest neighbor distances for all points:
  2 $$\begin{aligned} r_{obs} =\frac{1}{k}\sum \limits _{i=1}^k {d_i }, \end{aligned}$$
  where *k* is the number of nearest neighbor points, and $d_i$ is the nearest neighbor distances. The *k* can be specified before interpolating.
3. Obtain the nearest neighbor statistic $R\left( {S_0 } \right)$ by:
  3 $$\begin{aligned} R\left( {S_0 } \right) =\frac{r_{obs} }{r_{\exp } }, \end{aligned}$$
  where $S_{0 }$ is the location of an interpolated point.

### *Step 2*

 Normalize the $R({S_0})$ measure to $\mu _R$ such that $\mu _R$ is bounded by 0 and 1 by a fuzzy membership function:

4 $$\begin{aligned} \mu _R =\left\{ {\begin{array}{ll} 0&{}\quad R\left( {S_0 } \right) \le R_{\min } \\ 0.5-0.5\cos \left[ {\frac{\pi }{R_{\max } }\left( {R\left( {S_0 } \right) -R_{\min } } \right) } \right] &{}\quad R_{\min } \le R\left( {S_0 } \right) \le R_{\max } \\ 1&{}\quad R\left( {S_0 } \right) \ge R_{\max}\end{array}} \right., \end{aligned}$$

where $R_{\min }$ or $R_{\max }$ refers to a local nearest neighbor statistic value (in general, the $R_{\min }$ and $R_{\max }$ can be set to 0.0 and 2.0, respectively).

### *Step 3*

 Determine the distance-decay parameter $\alpha$ by mapping the $\mu _{R}$ value to a range of $\alpha$ by a triangular membership function that belongs to certain levels or categories of distance-decay value; see Eq. (5).

5 $$\begin{aligned} \alpha \left( {\mu _R } \right) =\left\{ {{\begin{array}{ll} {\alpha _1 } &{}\quad {{0.0}\le \mu _R \le {0.1}} \\ {\alpha _1 \left[ {1-5\left( {\mu _R -0{.}1} \right) } \right] +5\alpha _2 \left( {\mu _R -0{.}1} \right) } &{}\quad {{0.1}\le \mu _R \le {0.3}} \\ {5\alpha _3 \left( {\mu _R -0{.}3} \right) +\alpha _2 \left[ {1-5\left( {\mu _R -0{.}3} \right) } \right] } &{}\quad {{0.3}\le \mu _R \le 0{.}5} \\ {\alpha _3 \left[ {1-5\left( {\mu _R -0{.5}} \right) } \right] +5\alpha _4 \left( {\mu _R -0{.}5} \right) } &{}\quad {{0.5}\le \mu _R \le {0.7}} \\ {5\alpha _5 \left( {\mu _R -0{.7}} \right) +\alpha _4 \left[ {1-5\left( {\mu _R -0{.7}} \right) } \right] } &{}\quad {{0.7}\le \mu _R \le {0.9}} \\ {\alpha _5 } &{}\quad {{0.9}\le \mu _R \le {1.0}} \\ \end{array} }} \right. , \end{aligned}$$

where the $\alpha _{1}, \alpha _{2}, \alpha _{3}, \alpha _{4}, \alpha _{5}$ are the assigned to be five levels or categories of distance-decay value.

After determining the parameter $\alpha$, the desired prediction value of each interpolated point can be obtained via the weighting average. This stage is the same as that in the standard IDW.

## The improved GPU-accelerated AIDW method

This section will briefly introduce the considerations and strategies in the development of the improved GPU-accelerated AIDW interpolation algorithm.

### Overview and basic ideas

The basic and most interesting concept behind the AIDW method is as follows. It attempts to determine adaptively the power parameter $\alpha$ according to the spatial distribution pattern of each interpolated point. In AIDW algorithm, the spatial distribution pattern is considered as the distribution density of several nearest neighboring data points locating around an interpolated point, which can be roughly measured by using the average distance from those neighboring data points to the interpolated point.

In our previous work, we present a straightforward, easy-to-implement, and suitable for GPU-parallelization algorithm to find the *k* nearest neighboring data points of each interpolated point. Assuming there are *n* interpolated points and *m* data points, for each interpolated point we carry out the following steps (Mei et al. 2015):

- *Step 1* Calculate the first *k* distances between the first *k* data points and the interpolated points;
- *Step 2* Sort the first *k* distances in ascending order;
- *Step 3* For each of the rest ($$m-k)$$ data points,

1. Calculate the distance *dist*;
2. Compare the *dist* with the *k*th distance: if *dist* < the *k*th distance, then replace the *k*th distance with the *dist*
3. Iteratively compare and swap the neighboring two distances from the *k*th distance to the first distance until all the *k* distances are newly sorted in ascending order.

The major advantage of the above algorithm is that it is simple and easy to implement. Obviously, there is no need to utilize any complex space partitioning data structures such as various types of *trees*. In contrast, only arrays for storing distances and coordinates are needed. Also, we find the desired nearest neighbors without the use of explicit sorting algorithms such as binary search. In general, most sorting algorithms are computationally complex and not suitable for entirely being invoked within a single GPU thread.

The most obvious shortcoming of the above algorithm for finding nearest neighboring data points is the computational inefficiency that is due to the global search for nearest neighbors. In that algorithm, the first *k* distances are calculated and recorded; and then the distances to the rest points are calculated and then compared with those first *k* distances. The above procedure obviously needs a global search, which is not computationally optimal. One of the frequently used optimization strategies is to perform a local search by filtering those data points and distances that are not needed to be considered.

In this work, we focus on improving our previous GPU-accelerated AIDW algorithm by using a fast *k*NN search algorithm. Our considerations and basic ideas behind developing the efficient *k*NN search algorithm are as follows:

1. Create an even grid to partition the planar region that encloses the projected positions of all data points and interpolated points;
2. Distribute all the data points and interpolated points into the grid and record the locations;
3. Perform a *local* and fast search within the grid to find the nearest neighboring data points for each interpolated point.

After obtaining the average distance of those neighboring data points, the adaptive power parameter $\alpha$ will be determined according to the average distance. Finally, the desired prediction value for each interpolated point can be obtained via weighting average using the parameter $\alpha$.

**Fig. 1:**
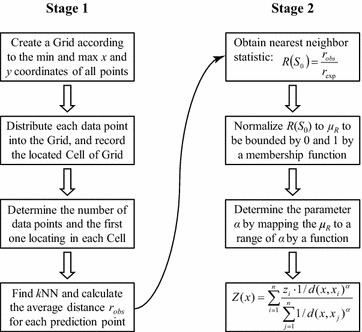
Process of the improved GPU-accelerated AIDW interpolation algorithm

In summary, the improved GPU-accelerated AIDW algorithm is mainly composed of two stages: (1) the *k*NN search and average distances calculation, and (2) the determination of adaptive power parameter and prediction value by weighted interpolating; see Fig. 1.

### Stage 1: *k*NN search

The workflow of the stage of *k*NN search is listed in Fig. 1. In this section, more descriptions on this stage will be presented.

#### Creating an even grid

**Fig. 2:**
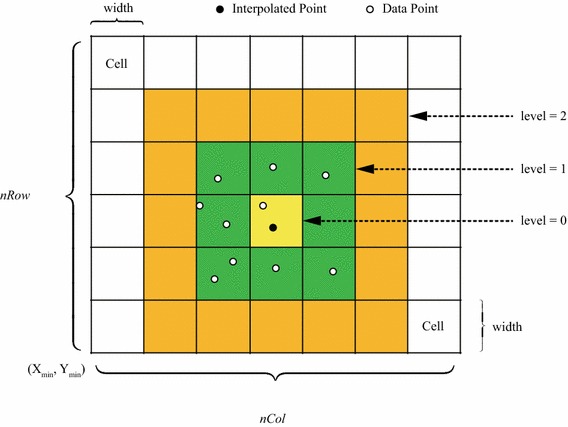
The creation of an even grid according to the minimum and maximum coordinates of all the data points and interpolated points

The even grid is a simple type of data structure for space partitioning, which is composed of regular cells such as squares or cubes; see an example of planar grid illustrated in Fig. 2. Compared to other efficient but complex space partitioning data structures such as the *k*-d tree, the even grid is much easier to create and search objects. In this work, we use a planar even grid to partition all data points to speed up the *k*NN search via local search.

The building of an even planar grid is straightforward. We first calculate or specify the width of the square cell, then determine the planar rectangular region for partitioning according to the minimum and maximum *x* and *y* coordinates of all points, i.e., obtain the length and width of the rectangle. After that, the numbers of rows and columns of the grid can be quite easily determined by dividing the rectangle.

#### Distributing data points into cells

The distribution of each data point is to find out that in which grid cell the data point locates. Since each grid cell can be located and recorded using its row and column indices, the distribution of each data point is in fact to obtained the row and column indices of the cell in which it locates.

This procedure can also be quite easily performed. First, the differences between the coordinates of the data points and the minimum coordinates of all cells are calculated; then the indices of row and column can be obtained by dividing the above differences with the cell width.

**Fig. 3:**
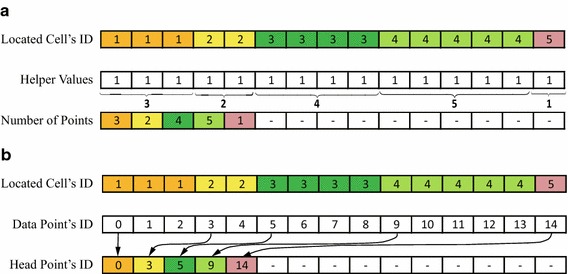
Demonstration of determining the number of data points distributed in each cell and the index of the head point. **a** the number of points; **b** the index of the head point

**Fig. 4:**
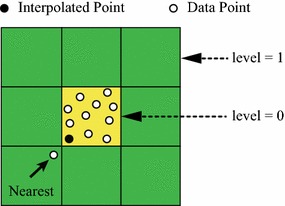
An example for demonstrating the failure of finding exact nearest data points for an interpolated point

#### Determining data points in each cell

The most important and basic idea behind utilizing a space partitioning is to perform a local search within local regions rather than a global search. When searching nearest neighbors, it is computationally optimal to first search approximate nearest neighbors within several local cells and then to find the exact nearest neighbors by filtering undesired points.

Since the local search is operated within cells, it is thus needed to determine that which data points locate inside a specific cell. In other words, it is needed to know the number and the indices of those data points locating in the same cell. Moreover, the layout for storing the number and indices should be carefully handled.

For each grid cell, to store the above-mentioned number and indices of those data points locating in the same cell, in general, a dynamic array of integers needs to be allocated. In the traditional CPU computing, the allocation and operations of dynamic arrays are easy-to-implement and computationally inexpensive. However, in GPU computing, it is no longer easy to implement or computationally cheap. This is due to the following two reasons. (1) In GPU computing the programming model such as CUDA cannot support the allocation and operations of dynamic arrays/containers like vector and list in C++ STL (Standard Template Library); and (2) the allocation of a large-enough static array of integers, e.g., int index[1000], for storing the indices of data points within each GPU thread is not memory efficient.

Due to the above reasons, we design an optimal layout for storing the number and indices of data points. Our basic idea is as follows. If the indices of those points locating inside the same cell are stored in a continuous segment/piece of integer values, then we only need to know the address of the first point in the segment and the number of points in the same segment (i.e., the size of the segment).

In this case, for each cell, we can only use two integer values to record the number and the indices of those data points that locate in the same cell. One integer is used to hold the number, and the other is used to record the address of the head/first point in each segment. The above two values can be very efficiently determined in a parallel fashion.

Before determining the number and indices of data points locating in the same cell, those data points should be recorded continuously. Since we have obtained the index of the cell in which each data point locates, if we sort all data points according to their corresponding cell indices in ascending order, then those data points locating in the same cell can be gathered in a continuous segment. This sorting procedure is suited to be parallelized on the GPU.

The number of data points locating in the same cell is determined using *segmented* parallel reduction. As described above, after sorting all data points according to cell indices, all data points are stored in a group of segments; each segment is flagged with the cell index, and contains the indices of data points locating in the same cell. The number of data points locating in the same cell can be achieved by performing a reduction for each segment; see Fig. 3a. Similarly, the head index of the first point of each segment can be obtained using segmented parallel scan; see Fig. 3b.

#### Searching nearest neighbors

In this work, a space-partitioning data structure, the even grid, is employed to enhance the *k*NN search algorithm. The most important and basic idea behind utilizing the space partitioning is to perform a local search within local regions rather than a global search. This idea is quite effective in practice for that the number of points that are needed to find and compare can be significantly reduced, and therefore, the computational efficiency can be improved.

The process of *k*NN search for each interpolated point can be summarized as follows.

- *Step 1* Locate the interpolate point into the even grid
- *Step 2* Determine the level of cell expanding
- *Step 3* Find the nearest neighbors within the local region
- *Step 4* Calculate the average distance

The locating of each interpolated point into the previously created planar grid is quite straightforward. Since each grid cell can be located and recorded using its row and column indices, the distribution of each interpolated point is in fact to obtained the row and column indices of the cell in which it locates. First, the differences between the coordinates of the interpolated point and the minimum coordinates of all cells are calculated; then the indices of row and column can be obtained by dividing the above differences with the cell width.

The determining of the level of cell expanding is in fact to determine the region of cells in which the local nearest neighbors search should be carried out; see three levels of cell expanding in Fig. 2. In *k*NN search, the number of nearest neighbors, *k*, is typically pre-specified; and obviously, the number of data points locating in the local cells must be larger than the number *k*. Thus, the level of cell expanding can be iteratively determined by comparing the number of currently found data points with the number *k*. For example, when the *k* is specified as 15, and within the first level of local cells there are only ten data points, and thus the level 1 needs to expand to level 2. Similarly, if only 14 data points can be found within the second level of local cells, the level needs to be further expanded to 3. This procedure is iteratively repeated until enough data points have been found.

##### *Remark*

Note that after iteratively determining the level of cell expanding, for example, level 3, the final level of cell expanding needs to increase with 1, i.e., level 4. This is due to the following reason. Without expanding additional one level, the nearest neighbors found in the initial level of local cells may not the desired exact *k* nearest neighbors; see the marked data point in Fig. 4. When $k = 10,$ the determined level of cell expanding is 0 (i.e., the yellow region). However, the marked data point is obvious one of the nearest neighbors of the only interpolated point because it is much nearer to the interpolated point than several data points locating in the yellow region. This demonstrates that: without expanding additional one level, incorrect/undesired nearest neighboring data points are probably found; and several of the expected nearest neighboring data points may not able to be found.

The *k*NN search in the local cells is, in fact, to further find exact nearest neighbors by filtering some undesired points. We first allocate an array with the size of *k* for storing distances, and initiate all distances to 0. Then for each of those data points locating in the local cells, we calculate the distance *dist*, and compare the *dist* with the *k*th distance; and if *dist* is smaller than the *k*th distance, then replace the *k*th distance with the *dist*; after that, we iteratively compare and swap the neighboring two distances from the *k*th distance to the first distance until all the *k* distances are newly sorted in ascending order; see Mei et al. (2015) for more details.

After finding the nearest neighbors of each interpolated point, the distances between each nearest neighbor and the interpolated point can be calculated; and finally, the desired average distance can be obtained.

**Fig. 5:**
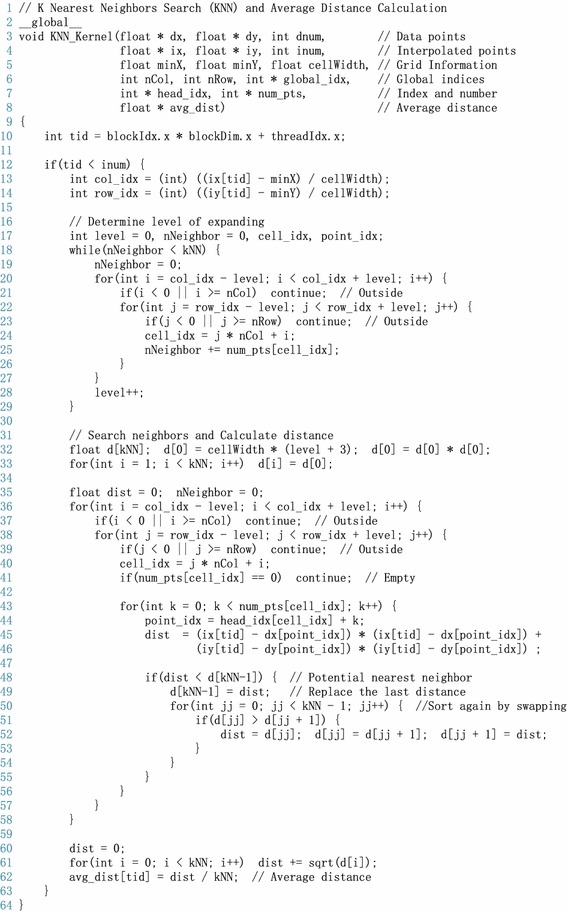
A CUDA kernel of the *k*NN search

**Fig. 6:**
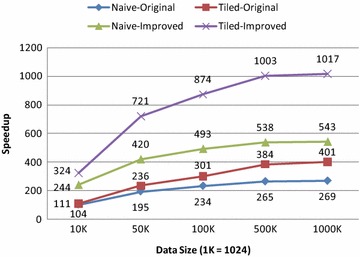
Speedups of the improved and the original GPU-accelerated AIDW algorithms over the serial AIDW algorithm

**Fig. 7:**
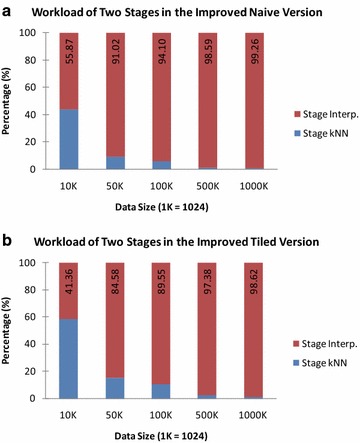
Workload of the two stages in the improved GPU-accelerated AIDW algorithm. **a** Naive version; **b** tiled version

**Fig. 8:**
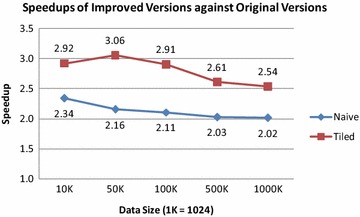
Speedups of the improved GPU-accelerated AIDW algorithm over the original algorithm for both the naive version and tiled version

**Fig. 9:**
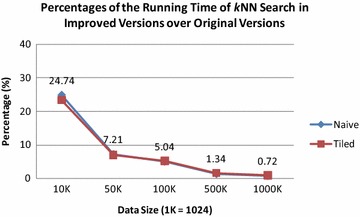
Percentages of the running time of *k*NN search in the improved algorithm over the original algorithm

### Stage 2: weighted interpolating

According to the principle of the AIDW interpolation algorithm, it is perfect that a single GPU thread can take the responsibility to calculate the prediction value of an interpolated point. For example, assuming there are *n* interpolation points that are needed to be predicted their values such as elevations, and then it is required to allocate *n* threads to calculate the desired prediction values for all those *n* interpolated points concurrently.

In GPU computing, data access on the shared memory is inherently much faster than that on the global memory; therefore, any choices to replace global memory access by shared memory access should be utilized. Due to the fact that the shared memory residing in the GPU is limited per SM (Stream Multiprocessor), a common optimization strategy called “tiling” is frequetnly used to handle the above problem, which divides the data stored in global memory into pieces called tiles so that each tile fits into the size of shared memory.

The above-mentioned strategy of “tiling” is also employed to enhance the GPU-accelerated AIDW algorithm. First, the coordinates of data points are loaded from the global memory into the shared memory. Then, all threads within the same thread block are able to concurrently access the coordinates currently residing in the shared memory. With the utilization of the strategy of “tiling”, the accesses to the global memory can be obviously reduced; and performance gains are expected to be achieved.

## Implementation details

As introduced in the above section, the improved GPU-accelerated AIDW interpolation algorithm is mainly composed of two stages, i.e., the *k*NN search stage and the weighted interpolating stage. In this section, we will describe some implementation details on the above two stages.

### Stage 1: *k*NN search

#### Creating an even grid

An even grid is composed of a group of grid cells, and in this work, each grid cell is a square. The creation of an even grid is in fact to determine the position of the grid, the size of the cell, and the distribution layout of the cells. In our algorithm, an even planar grid is created to cover the planar region in which the projected positions of all data points and interpolated points locate.

We first obtain the minimum and maximum coordinates of all the data points and interpolated points using the parallel reduction thrust::minmax_element() provided by the library *Thrust* (Bell and Hoberock 2012), and calculate the differences between those minimum and maximum coordinates in *x*- and *y*- direction. After approximately determining the planar region, we then calculate the length of interval cellWidth, i.e., the width of a square cell, according to Eq. (1). After that, the number of rows and columns of grid cells can be easily calculated.

#### Distributing data points into cells

After creating the even grid, the subsequent step is to distribute all the data points into the grid. This procedure can be naturally parallelized since the distributing of each data point can be performed independently. Assuming there are *m* data points, we allocate *m* GPU threads to distribute all the data points. Each thread is responsible for calculating the position of one data point locating in the grid, i.e., to determine the index of the cell where the data point locates.

A cell in a grid can be exactly positioned according to the indices of row and column, i.e., int col_idx, row_idx. Also, the position of each grid can be found according to its global index that can be calculated using the simple transformation, global_idx = row_idx * nCol + col_idx.

The above transformation formulation can be used to transform a two-dimensional index of each grid cell to a unique one-dimensional index. Obviously, this transformation can be easily transformed back. The reasons why we carry out the transformation are as follows. First the memory requirement is reduced since only one array of integers is needed to be stored; and the second is that sorting with using one value as the key is much faster than that with two values as keys.

To obtain the indices and numbers of those data points locating in each cell, an effective solution is to store those data points that locate in the same cell continuously. Then, operations on the continuous pieces of data (i.e., segments) can be very efficient; see more descriptions in the closely subsequent section.

#### Determining data points in each cell

In the stage of the *k*NN search, our objective is to find *k* nearest neighboring data points for each interpolated point. The *k*NN search for each interpolated point is locally performed within several grid cells. The first requirement is to determine how many and which data points locate in each grid cell. More specifically, we need to know the indices and the number of those data points locating in each grid cell. We obtain this simply by using parallel reduction and scan; see our ideas illustrated in Fig. 3.

Before carrying out the parallel reduction and scan, those data points that locate inside the same cell should be stored continuously. This requirement can be fulfilled by utilizing a parallel sort with the use of the global index of cells as keys. The parallel sort is realized by using the corresponding parallel primitive provided by the powerful library *Thrust*, thrust::sort_by_key(keys, values).

Note that those data points locating in the same cell are stored continuously, and if we know the number of data points locating in the same cell, then we only to know the first address of the first data point; and each of the rest data points can be referenced according to the address of the first point and its local position. This idea is quite similar to the reference of any value/element in an array.

Then, the parallel reduction and scan are also performed by using the primitives provided by *Thrust*. We also use the global index of cells as the keys for ***Segmented*** reduction and scan. The motivation why we use the segmented reduction and scan rather than the global reduction and scan is that in the current step we only need to operate on the data points locating in the same cell; and those data points locating in the same cell have been stored continuously and marked using the global index of cell as flags; see Fig. 3.

The number of those data points locating in the same cell is obtained by using the primitive thrust::reduce_by_keys(); and the index of the first/head point of each segment of data points are found using thrust::unique_by_keys(). As illustrated in Fig. 3, a helper array of constant integers is additionally used to count the number of data points stored in the same piece/segment.

#### Searching nearest neighbors

The finding of *k* nearest neighboring data points for each interpolated points can be inherently parallelized. Assuming there are *n* interpolated points, and we allocate *n* threads to search the nearest neighbors for all the interpolated points. Each thread is invoked to find the nearest neighbors for only one interpolated point.

Within each thread, we first distribute the interpolated point into the created grid by calculating its row index and column index; see lines 13–14 in Fig. 5. Then we determine the region of the local cells by approximately calculating the level of expanding according to the number of data points; see lines 16–29 in Fig. 5. Note that currently those data points locating in the determined local cells are the ***Approximate*** nearest neighbors of the interpolated points. After that, we further find the ***Exact*** nearest neighbors by filtering those approximate nearest neighbors by inserting and swapping; see lines 31–58 in Fig. 5. Finally, the desired average distance between the exact nearest neighboring data points and the target interpolated point is calculated.

Note that there is a remarkable implementation detail. When finding the nearest neighbors according to the *Euclidean* distances between points, we do not use the real distance value but the square value of the distance. This is because in GPU computing the calculation of square root is quite computationally expensive; and any choice to avoid the use of calculating square root should be exploited. Thus, we calculate the square root in the last step of computing the average distance, rather in the step of searching nearest neighbors.

### Stage 2: weighted interpolating

This subsection will present the details on implementing the interpolating stage in the GPU-accelerated AIDW algorithm. We implement two versions: the *naive* version and the *tiled* version, by employing the data layout Structure-of-Arrays (SoA) only. Both the naive and the tiled implementations developed in this work are the same as those corresponding implementations presented in our previous work (Mei et al. 2015).

#### Naive version

In this version, the global memory and registers on GPU architecture are employed without exploiting the shared memory. The input data and the output data are stored in the global memory. Assuming that there are *m* data points used to evaluate the prediction values for *n* interpolation points, we allocate *n* threads to parallelize the interpolating.

Since that after invoking the *k*NN kernel, we have obtained the average distance, i.e., the $r_{obs}$ defined in Eq. (2), thus in this stage each thread is only responsible for computing the $r_{\exp }$ and $R\left( {S_0 } \right)$ according to the Eqs. (1) and (3). After that, the $R\left( {S_0 } \right)$ measure is normalized to $\mu _R$ such that $\mu _R$ is bounded by 0 and 1 by a fuzzy membership function; see Eq. (4). Finally, the power parameter $\alpha$ is determined by mapping the $\mu _{R}$ values to a range of $\alpha _{ }$ by a triangular membership function; see Eq. (5).

After adaptively determining the power parameter, the desired prediction value of each interpolated point can be achieved by weighting average. This step of calculating the weighting average is the same as that in the standard IDW method.

#### Tiled version

The workflow of the tiled version is the same as that of the naive version. However, in the tiled version the shared memory is exploited to improve the computational efficiency, while in the naive the shared memory is not utilized.

When implementing the tiled version, the tile size (i.e., the number of tiles) is simply specified as the same as the block size (i.e., the number of blocks within a grid). Each thread within the grid is responsible to (1) transferring the coordinates of only one data point from the global memory to the shared memory, and (2) calculating the distances and weights to those data points currently residing in the shared memory.

When all the threads within a thread block complete the calculating of partial distances and weights, the subsequent wave of points’ coordinates is transferred from the global memory to the shared memory again. This new piece of data is employed to compute the current wave of partial distances and corresponding weights.

When finishing the calculation of all waves of partial distances and weights, each thread is invoked to accumulate all the weights and weighted values into two registers. At last, the desired prediction value of an unknown point, i.e., the weighted average, is computed and then written to the global memory.

## Results and discussion

### Experimental environment and testing data 

In this work, we focus on improving our previous GPU-accelerated AIDW algorithm by utilizing a fast *k*NN search method. We refer our previously developed GPU-accelerated AIDW algorithm as the *original* algorithm, and the presented algorithm in this work as the *improved* algorithm.

To evaluate the computational efficiency of the improved algorithm, we have carried out five groups of experimental tests on a laptop computer which features with an Intel Core i7 CPU (2.40GHz), 4.0 GB RAM memory, and a GeForce GT730M card. All the experimental tests are executed on OS Windows 7 Professional (64-bit), Visual Studio 2010, and CUDA v7.0.

Two versions of the improved GPU-accelerated AIDW, i.e., the naive version and the tiled version, are implemented using the SoA layout and evaluated on single precision. In contrast, the CPU version of the AIDW implementation is tested on double precision; and all results of this CPU version presented in our previous work (Mei et al. 2015) are directly accepted to be used as the baseline. The efficiency of all GPU implementations is benchmarked by comparing to the baseline results.

When evaluating the execution time of GPU implementations, the overhead spent on transferring the input data (i.e., the coordinates of data points and interpolated points) from the host side to the device side and transferring the results from the device side to the host side is considered. However, the time spent on generating the test data is not included.

The input of the AIDW interpolation is the coordinates of data points and interpolated points. The efficiency of the CPU and GPU implementations may differ due to different sizes of input data. However, the research objective in this work is to improve our previous GPU-accelerated AIDW algorithm using fast *k*NN search; thus, we only consider a particular situation where the numbers of interpolated points and data points are identical.

All the testing data including the data points and interpolated points are randomly generated within a square. We design five groups of sizes, i.e., 10K, 50K, 100K, 500K, and 1000K, where one K represents the number of 1024 (1K = 1024). Five tests are performed by setting the numbers of both the data points and interpolated points as the above five groups of sizes.

### Performance of the improved GPU-accelerated AIDW algorithm

#### Computational efficiency

We evaluate the computational efficiency of the improved GPU-accelerated AIDW algorithm with the use of five groups of testing data. The running time and the GFLOPS are listed in Table 1 and Table 2, respectively. Note that, to compare with the original GPU-accelerated algorithm, we have also listed the execution time of the original algorithm in Table 1; and these experimental results of the original algorithm are directly derived from our previous work (Mei et al. 2015).

**Table 1:** Execution time (/ms) of CPU and GPU versions of the AIDW algorithm on single precision

Version	Data size (1K = 1024)				
	10K	50K	100K	500K	1000K
CPU/serial	6791	168,234	673,806	16,852,984	67,471,402
Original naive version	65.3	863	2884	63,599	250,574
Original tiled version	61.3	714	2242	43,843	168,189
Improved naive version	27.9	400	1366	31,306	124,353
Improved tiled version	21.0	233	771	16,797	66,338

**Table 2:** GFLOPS of the original and the improved GPU-accelerated AIDW algorithms when the data size is set as 1000K (1K = 1024)

Version	GFLOPS
Original naive version	51.81
Original tiled version	107.12
Improved naive version	100.95
Improved tiled version	192.34

We have also calculated the speedups of our improved GPU-accelerated AIDW algorithm against the corresponding serial algorithm (i.e., the CPU version listed in Table 1) and see Fig. 6. The results indicate: (1) the highest speedups achieved by the naive version and the tiled version can be up to 543 and 1017, respectively; and (2) the tiled version is always faster than the naive version. The GFLOPS listed in Table 2 also demonstrates that the tiled version is always faster than the naive version for both the original and the improved GPU-accelerated AIDW algorithms.

#### Comparison of the improved naive version and tiled version

As observed from the experimental tests, the tiled version of the improved algorithm is about 1.33–1.87 times faster than the naive version. This behavior is due to the fact that the stage of interpolating in the tiled version is much more computationally efficient than that in the naive version; see the execution time of the interpolating stage in Table 3.

As described in “The improved GPU-accelerated AIDW method” section, the improved algorithm includes both the naive version and tiled version, which can be divided into two major stages: i.e., the stage of *k*NN search and the stage of weighted interpolating. The first stage in the above two versions are the same, while the second stage differs.

In the stage of interpolating of the tiled version, the benefit of the use of shared memory is exploited, while in the naive version it is not. For this reason, the interpolating stage in the tiled version executes about 1.79–1.89 times faster than that in the naive version. Thus, the entire tiled version is more efficient than the naive version. This is also demonstrated according to the GFLOPS of the naive version and tiled version; see Table 2.

**Table 3:** Execution time (/ms) of the stage of *k*NN search and the stage of weighted interpolating in the improved GPU-accelerated AIDW algorithm

Stage	Data size (1K = 1024)				
	10K	50K	100K	500K	1000K
*k*NN search (both versions)	12.3	36	81	440	917
Weighted interpolating (improved naive version)	15.6	364	1286	30,866	123,437
Weighted interpolating (improved tiled version)	8.7	197	691	16,357	65,421

#### Workload between the stages of *k*NN search and weighted interpolating

There are two major stages in the improved GPU-accelerated AIDW algorithm. To understand the efficiency bottleneck for further optimizations in the future, we in particular record the execution time for the stages of *k*NN search and weighted interpolating separately; see Table 3. In addition, we have also evaluated the workload percentage between the above two stages in both the naive version and tiled version; see Fig. 7.

We have found the computational cost spent in the stage of *k*NN search is much less than that in the stage of the weighted interpolating. Moreover, with the increase of the size of testing data, the weight of the running time cost in the stage of *k*NN significantly decreases; and it even reduces to about one percentage. This observation indicates that most overhead in both the naive version and the tiled version is spent in the stage of weighted interpolating rather than the *k*NN search. Therefore, further optimizations may need to be employed to improve the efficiency of the weighted interpolating.

### Comparison with the original GPU-accelerated AIDW algorithm

In “Experimental environment and testing data” section, the efficiency of the improved GPU-accelerated AIDW algorithm has been compared with that of the original serial AIDW algorithm (Tables 1, 2); and it was found that the proposed improve algorithm can obtain quite satisfactory speedups. In this subsection, the present improved GPU-accelerated AIDW algorithm will be benchmarked with the original GPU-accelerated AIDW algorithm introduced in Mei et al. (2015).

The speedups of the improved GPU-accelerated AIDW algorithm over the original algorithm are illustrated in Fig. 8. The results show that the improved naive version and tiled version are at least 2.02 and 2.54 times faster than the original naive version and tiled version, respectively. This also indicates that significant performance gains have been achieved by improving the original algorithm using fast *k*NN search.

The major difference between the original algorithm and the improved algorithm is the use of different *k*NN search approaches. We attempt to explain the reason why significant performance gains have been achieved by analyzing the impact of different *k*NN search algorithm on the computational efficiency.

First, we obtain the computational time of the *k*NN search in the original algorithm by subtracting the time spent in the stage of weighted interpolating from the total execution time; see Table 4. Note that, the execution time cost in the stage of weighted interpolating is directly derived from the improved algorithm. This is because (1) the weighted interpolating in both the original algorithm and the improved algorithm is the same; and (2) the running time of the weighted interpolating can be separately measured in the improved algorithm, while in contrast it is unable to accurately evaluate the execution time specifically for the weighted interpolating in the original algorithm.

**Table 4:** Execution time (/ms) of the stage of *k*NN search in the original and the improved GPU-accelerated AIDW algorithm

Version	Data size (1K = 1024)				
	10K	50K	100K	500K	1000K
Original naive version	49.7	499	1598	32733	127137
Original tiled version	52.6	517	1551	27486	102768
Both of improved versions	12.3	36	81	440	917

Second, we calculate the percentages of the running time of the *k*NN search in the improved algorithm over that in the original algorithm; see Fig. 9. We have found in both the naive version and the tiled version, the execution time of the *k*NN search in the improved algorithm is much less than that in the original algorithm, for example, less than one percentage for about one million points. This suggests the use of fast *k*NN search approach can significantly improve the efficiency of the entire GPU-accelerated AIDW interpolation algorithm (see Additional file 1).

## Conclusion

In this work, we have presented an efficient AIDW interpolation algorithm on the GPU by utilizing a fast *k*NN search method. The presented algorithm is composed of two major stages, i.e., the *k*NN search and weighted interpolating, and is developed by improving a previous GPU-accelerated AIDW algorithm with the use of fast *k*NN search. The *k*NN search is carried out based upon an even grid, and is capable of finding exact nearest neighbors very fast for each interpolated point. We have performed five groups of experimental tests to evaluate the performance of the improved GPU-accelerated AIDW algorithm. We have found: (1) the improved algorithm can achieve a speedup of up to 1017 over the corresponding serial algorithm for one million points; (2) the improved algorithm is at least two times faster than our previously developed GPU-accelerated AIDW algorithm; and (3) the utilization of fast *k*NN search can significantly improve the computational efficiency of the entire GPU-accelerated AIDW algorithm.

To benefit the community, all source code and testing data related to the presented AIDW algorithm is publicly available. In the future, further improvements in the computational efficiency are planed to be achieved by adopting different algorithm mapping strategies on a GPU and multi-GPU architecture (Cuomo et al. 2014).

## Additional file

10.1186/s40064-016-3035-2 cudaAIDW_*k*NN: Source code of the improved GPU-accelerated adaptive IDW algorithm.
